# Histopathological and immunohistochemical evaluation of cellular response to a woven and electrospun polydioxanone (PDO) and polycaprolactone (PCL) patch for tendon repair

**DOI:** 10.1038/s41598-020-61725-5

**Published:** 2020-03-16

**Authors:** Mustafa Rashid, Jayesh Dudhia, Stephanie G. Dakin, Sarah J. B. Snelling, Roberta De Godoy, Pierre-Alexis Mouthuy, Roger K. W. Smith, Mark Morrey, Andrew J. Carr

**Affiliations:** 1 Nuffield Department of Orthopaedics, Rheumatology, and Musculoskeletal Sciences (NDORMS), University of Oxford, Oxford, UK; 2 NIHR Biomedical Research Centre, Oxford, UK; 3 Department of Clinical Sciences and Services, Royal Veterinary College, University of London, North Mymms, UK; 4 Department of Orthopedic Surgery, Mayo Clinic, Rochester, Minnesota USA

**Keywords:** Translational research, Musculoskeletal system

## Abstract

We investigated endogenous tissue response to a woven and electrospun polydioxanone (PDO) and polycaprolactone (PCL) patch intended for tendon repair. A sheep tendon injury model characterised by a natural history of consistent failure of healing was chosen to assess the biological potential of woven and aligned electrospun fibres to induce a reparative response. Patches were implanted into 8 female adult English Mule sheep. Significant infiltration of tendon fibroblasts was observed within the electrospun component of the patch but not within the woven component. The cellular infiltrate into the electrospun fibres was accompanied by an extensive network of new blood vessel formation. Tendon fibroblasts were the most abundant scaffold-populating cell type. CD45^+^, CD4^+^ and CD14^+^ cells were also present, with few foreign body giant cells. There were no local or systemic signs of excessive inflammation with normal hematology and serology for inflammatory markers three months after scaffold implantation. In conclusion, we demonstrate that an endogenous healing response can be safely induced in tendon by means of biophysical cues using a woven and electrospun patch.

## Introduction

Musculoskeletal disorders account for around 30% of all years lived with disability globally, and this is forecast to rise by 70% by 2030. Soft tissue disorders, such as tendon disease and tears, account for a significant component of this burden^1,2^. Rotator cuff tendon tears are the most common cause of shoulder pain in adults and patients with persistent symptoms, which often require surgical repair^3,4^. Unfortunately repair failure rates are high, around 40%, and can adversely affect patient outcomes^5^. Augmentation patch strategies have been proposed and are intended to provide mechanical support during repair healing and may also provide a scaffold for host tissue integration^6,7^. Artificial scaffolds have also been employed in other clinical areas such as hernia repair and ligament reconstruction. These patches may be made from synthetic polymers or based on modified human or animal extracellular matrix (ECM). A disadvantage of many of the currently available materials is that they do not accurately mimic the ultrastructure of tendon. Controlled clinical studies have shown limited success in terms of healing rate and improved pain and function^8,9^. Concerns exist about the use of ECM based scaffolds, including the risk of disease transmission, graft rejection, and sterile inflammation. Porcine small intestinal submucosa (SIS) patches have been shown to illicit an adverse foreign body immunological response. DNA has been identified in some patches, raising further serious safety concerns over the use of ECM based materials^10–12^. Concerns also exist about serious complications arising from nonabsorbable synthetic meshes which can erode surrounding tissues causing chronic pain, particularly when used in the pelvis [14].

In an attempt to address these issue we developed a synthetic biodegradable patch made of aligned electrospun fibers reinforced by a woven monofilament mesh^13^. The biomimetic properties of electrospun micro- and nanoscale fibers have been shown to provide biophysical cues to a range of musculoskeletal cell types^14–16^. *In vitro* studies demonstrate that electrospun scaffolds promote mesenchymal stem cell differentiation, tendon-derived cell attachment and cellular proliferation^17^. This spontaneous repair complicates evaluation of the efficacy of an augmentation strategy as the main objective is to induce a reparative cellular response. A recently reported validated ovine model of tendon injury which does not heal spontaneously is now available, allowing improved experimental design^18^.

The aims of this study were to investigate if a woven and electrospun PDO/PCL patch could safely induce a positive endogenous tendon fibroblast response and enable tendon repair in a non-healing large animal model.

## Materials and Methods

### Electrospinning

Polymer solutions were prepared by dissolving polydioxanone (PDO, 1.5–2.2 dl/g, Evonik, Essen, Germany) or polycaprolactone (PCL, Vornia, Dublin, Ireland) into 1, 1, 1, 3, 3, 3-hexafluoroisopropanol (HFIP, Apollo Scientific Limited, Cheshire, UK) at concentrations of 11% and 20% respectively (weight to volume ratios). Solutions were agitated at room temperature on a roller for at least 24 h to allow for complete dissolution of the polymers. Polymer solutions were electrospun with a temperature and humidity controlled electrospinning machine (IME technologies, Geldrop, Netherlands). PDO electrospun meshes were produced by electrospinning the PDO solution for 2 h onto a drum rotating at 2000 rpm with a voltage of 8.2 kV. PCL electrospun scaffolds in two configurations: (1) as aligned meshes by electrospinning the PCL solution for 2 h onto a drum rotating at 100 rpm and a voltage of 8.4 kV, (2) as thin grids by electrospinning onto a thin nickel mesh (Precision Micro, Birmingham, UK) for 15 mins at a voltage of 8.4 kV. For all experiments, the distance nozzle-collector was set at 20 cm, the temperature was maintained at 21 °C and humidity maintained at 30%. All samples were stored in a desiccator prior to use.

### Weaving

Twill weave structures were produced from PDO monofilaments (7/0, Riverpoint Medical, USA) using an industrial loom (model NH2 53, Jakob Müller AG, Frick, Germany).

### Patch assembly and cutting

Briefly, a PCL aligned mesh was sandwiched between the PDO woven component and a non-woven electrospun PDO layer. The components were then maintained together under a gentle pressure on a hot plate at 80 °C for 1–3 min to melt the PCL mesh (Tm = 65 °C), acting as an adhesive following solidification upon cooling. Similar steps were then used to attached an additional 6 layers of PDO aligned materials using the PCL grids. The number of layers of PDO sheets was selected to build a thickness of the electrospun component comparable to that of the woven component^13^. Following layering, samples were cut to shape (5 mm × 10 mm) using a hot wire cutter (Proxxon Thermocut 230/E, Wecker, Luxemburg) to melt edges and avoid fraying.

### Scanning electron microscopy (SEM)

Both sides of the patch (top electrospun layer and woven layer) were analyzed after gold coating using a SC7620 Mini Sputter Coater System (Quorum Technologies Ltd, East Sussex, UK) by scanning electron microscopy (SEM) using a Zeiss Evo LS15 Variable Pressure Scanning Electron Microscope (Carl Zeiss Microscopy GmbH, Oberkochen, Germany). Images were taken at 5000X magnification to allow a minimum of 30 fibers from 3 images to be measured for their diameter. The median fiber diameter was calculated in nanometers.

### Surgical procedures and macroscopic observations

**Figure 1:**
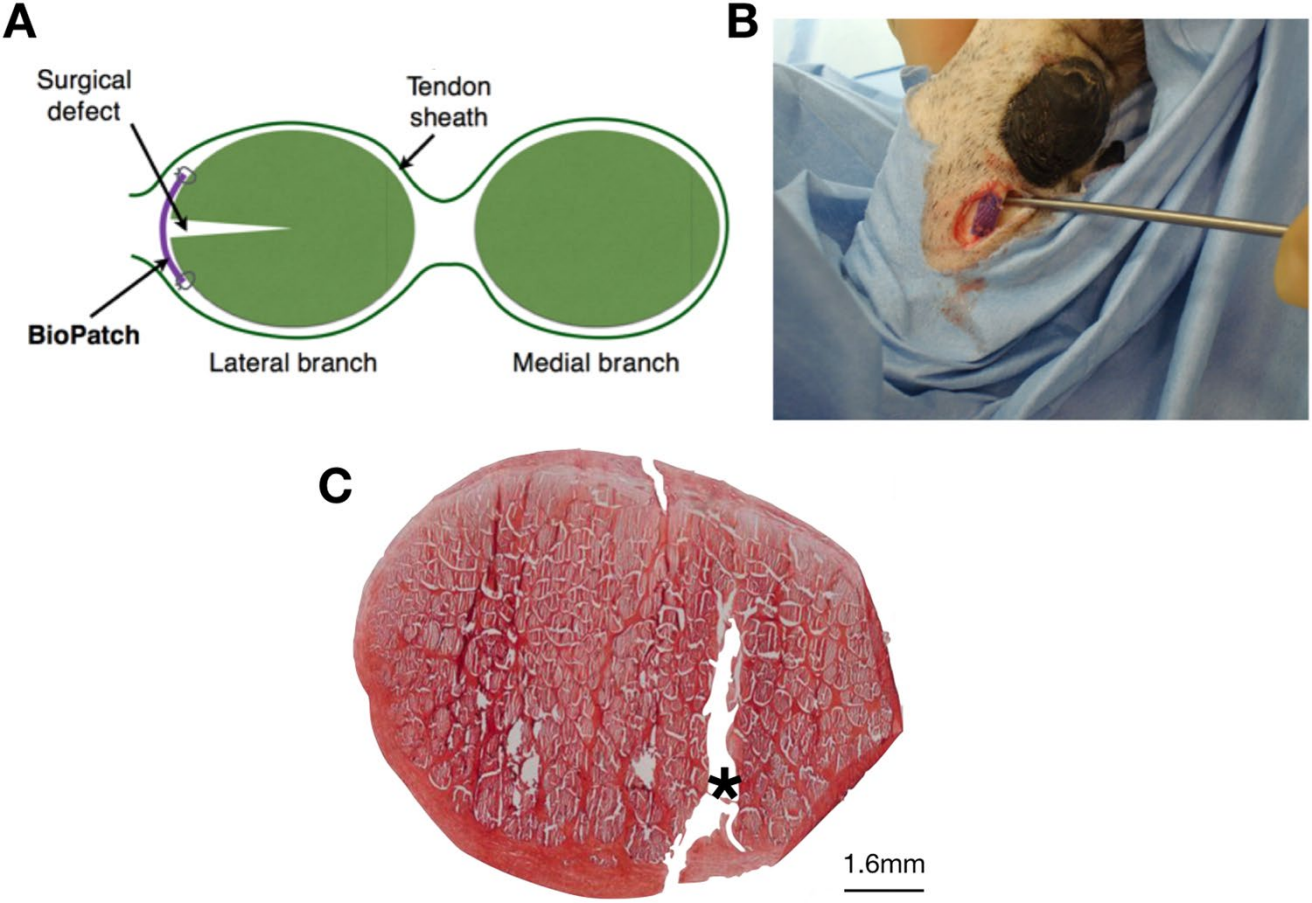
(**A**) Diagram shows cross-sectional view of the ovine deep digital flexor tendon (and enclosing digital flexor tendon sheath), demonstrating the surgical defect and the repair with patch (violet). (**B**) Photograph demonstrating patch applied over surgical defect on lateral branch of right forelimb deep digital flexor tendon. (**C**) Low magnification (2.5X) bright field microscopy image of H&E staining showing cross section of a typical non-healed DDFT tendon injury, 3 months after surgery without the use of a patch.

The study was performed under UK Home Office license (PPL70/6105) and with approval from the Ethics and Welfare Committee of the Royal Veterinary College. A sheep model of surgically induced tendon injury was used (Fig. 1)^18^. For this study, 8 female, skeletally mature, English mule sheep underwent surgery. All surgeries were performed under general anesthesia with intravenous injections of 2% xylazine (Rompun; Bayer Healthcare, Newbury, UK), ketamine (Ketaset; Fort Dodge Animal Health, Kansas, USA) and midazolam (Hypnovel; Roche, Burgess Hill, UK). Anaesthesia was maintained with ~2% isoflurane gas (IsoFlo, Abbott Labs, Maidenhead, UK) to effect. Briefly, after aseptic preparation, a 2.4 mm arthroscope was introduced into the lateral digital flexor tendon sheath immediately distal to the metacarpophalangeal joint. It was advanced into the proximal pouch of the sheath and a separate instrument portal made in the proximal sheath to allow the introduction of a hook knife. This was used to create a 10 mm long, 2 mm deep longitudinal cut in the deep digital flexor tendon. The instrument portal was then enlarged to allow open access to the tendon. The palmar annular ligament and the surrounding superficial digital flexor tendon was transected longitudinally to allow the deep digital flexor tendon (DDFT) at the site of the defect to be elevated using a hook. The patch scaffold was then applied over the tendon defect, and secured using four 4-0 PDO simple sutures in each corner of the patch (Figs. 1A,B). The skin incisions were closed and then the limb was bandaged before allowing the sheep to recover from general anesthesia. The sheep were initially housed in individual pens but then grouped in large pens which allowed free exercise from 1 week after surgery. They were assessed visually for lameness daily.

At 3 months, all sheep were euthanized by overdose of pentobarbitone at a dose of 1 ml/kg body mass. Necropsy was performed in all sheep according to standard operating protocols. During tendon harvest, all repairs were examined macroscopically for signs of tissue healing, residual inflammation, synovial fluid changes, and adhesion formation. The general appearance of the surrounding DDFT sheath and adjacent superficial digital flexor tendon (SDFT) was also recorded. Circumferential measurements proximal and distal to the metacarpophalangeal joint (MCPJ) were made to qualitatively assess swelling of the tendon repair site.

### Hematology and serum inflammatory markers

Blood samples for serum preparation were taken from all sheep pre-surgery and at 3 months post-surgery. Full blood count and serum inflammatory markers (serum amyloid A and fibrinogen) were performed.

### Histology and immunohistochemistry (IHC)

**Figure 2:**
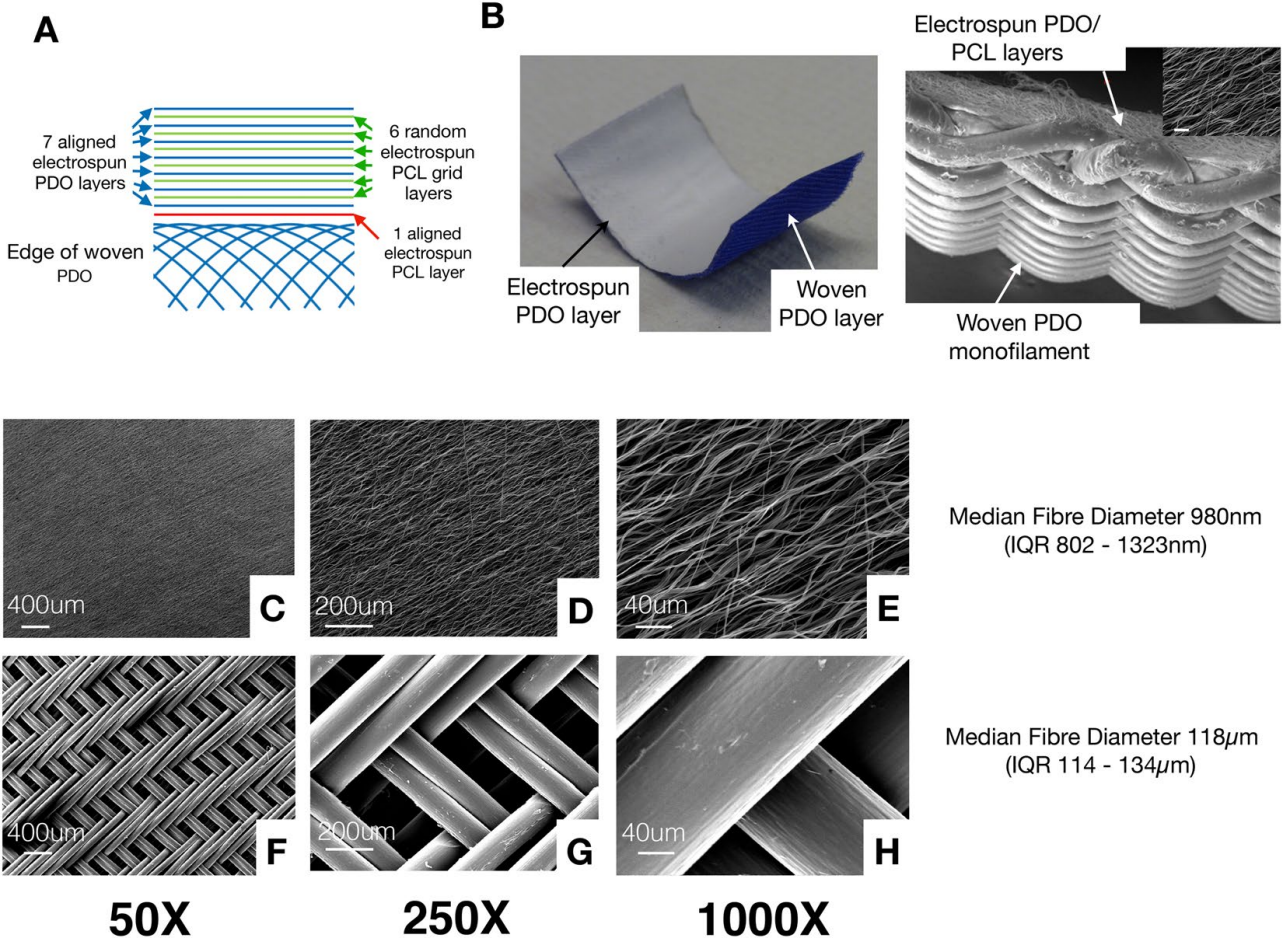
(**A**) Diagram representing different layers in the electrospun patch. Seven layers of aligned electrospun PDO fibers are sandwiched between 6 layers of thin PCL electrospun grids (acting as an adhesive) in an alternate manner forming the electrospun component of the patch. The electrospun component is bound to a woven PDO monofilament layer by a single layer of aligned electrospun PCL fibers. (**B**) Photograph of patch sample showing white electrospun PDO layer (facing tendon) and blue woven PDO layer. (**C**–**E**) Representative scanning electron microscopy (SEM) images showing electrospun layer. (**F**–**H**) Woven layer of the patch at various magnifications shown.

The operated lateral branch of the harvested DDFT was cut at the bifurcation and fixed in 10% formalin for 7 days. Tendon samples were then processed using a Leica ASP300S tissue processor and embedded in paraffin wax. 5–7 um sections were cut using a rotary RM-2135 microtome (Leica Microsystems Ltd, Milton Keynes, UK), and placed onto Surgipath X-tra Adhesive glass slides (Leica Microsystems Ltd, Milton Keynes, UK). Slides were baked at 60 °C for 30 minutes then 37 °C for 60 minutes. Histology with hematoxylin & eosin (H&E) staining was performed. Using a Zeiss AX10 inverted microscope with an Axiom HRc camera and Axiovision software (Zeiss, Cambridge, UK), bright field microscopy images were taken at high (100X) magnification in areas relating to the electrospun fiber zone (ES, shown in Fig. 2B), and normal tendon zone (NT, shown in Fig. 2D). The zone directly adjacent to the woven PDO layer can also be seen in Fig. 2C.

In 6 random images per field, per sample, histomorphometric features were used to record immune cell count (ICC), fibroblast cell count (FCC), and foreign body giant cell count (FBGCC). Vascularity was graded as follows:

0 = no signs of vascularity;

1 = occasional red blood cells (RBCs) seen, or one small vessel;

2 = several groups of RBCs, or more than one small blood vessel;

3 = extensive blood vessels, one or more very large vessel(s), or many RBCs seen in the field of view.

Immunohistochemistry was performed to identify immune cell types populating patch augmented tendons. Heat-induced, high pH epitope retrieval was performed using a PT Link machine (Dako Agilent Pathology Solutions, Santa Clara, USA). Antibody staining was performed using the EnVision FLEX visualization system with an Autostainer Link 48 (Dako) using anti-CD4 (a glycoprotein co-receptor on the surface of T-helper cells), anti-CD14 (a co-receptor found mainly on monocytes), anti-CD45 (a pan-leucocyte cell marker), and anti-TGFβ (a cytokine with pro-fibrotic effects) antibodies (further details are listed in Table 1). Antibody binding was visualized using FLEX 3,3′-diaminobenzidine (DAB) substrate working solution and hematoxylin counterstain (Dako) using the recommended manufacturer protocols. Images were acquired using the same microscope-camera system as above, at high magnification (100X) in several fields corresponding to areas relating to the electrospun fiber zone (ES), and normal tendon zone (NT). Immunopositive staining was determined and quantified as the proportion of DAB stained tissue (% of total area) divided by the number of nuclei present (to account for cellularity). All images taken in the electrospun fiber zone were compared to all images taken in the normal tendon zone, which was used as a control. Immunohistochemistry analysis was performed using a semi-automated software (CellProfiler, Broad Institute, Boston, MA). A custom algorithm was created to count number of nuclei and %DAB stain.

**Table 1:** Primary antibody details used in immunohistochemistry staining for patch samples.

Anti-	Source	Host Species	Clone/ID	Dilution
CD4	Biorbyt	Rabbit	Poly/orb182470	1: 1000
CD14	Abcam	Mouse	Tuk4/ab27545	1: 100
CD45	LS Bio	Mouse	1.11.32/LS-C187484	1: 200
TGFβ	LS Bio	Rabbit	Poly/LS-C352937	1: 150

### Statistical methods

Descriptive statistics included median values and 95% confidence intervals (CI). Comparisons between zones (ES and NT), using median values for immune cell count, fibroblast cell count, foreign body giant cell count, and vascularity were performed. A Mann Whitney U test was applied to determine the differences between fields (ES and NT) in the 4 categories (ICC, FCC, FBGCC, and vascularity) using Prism v7 (GraphPad, La Jolla, CA, USA). For the IHC analysis, a Mann Whitney U test was applied to detect differences between the median percentage DAB staining per nuclei between the electrospun fiber (ES) zones and normal tendon (NT) zones for all primary antibodies tested in all sheep. The level of statistical significance was set at p < 0.05.

## Results

### Scaffold morphology

**Figure 3:**
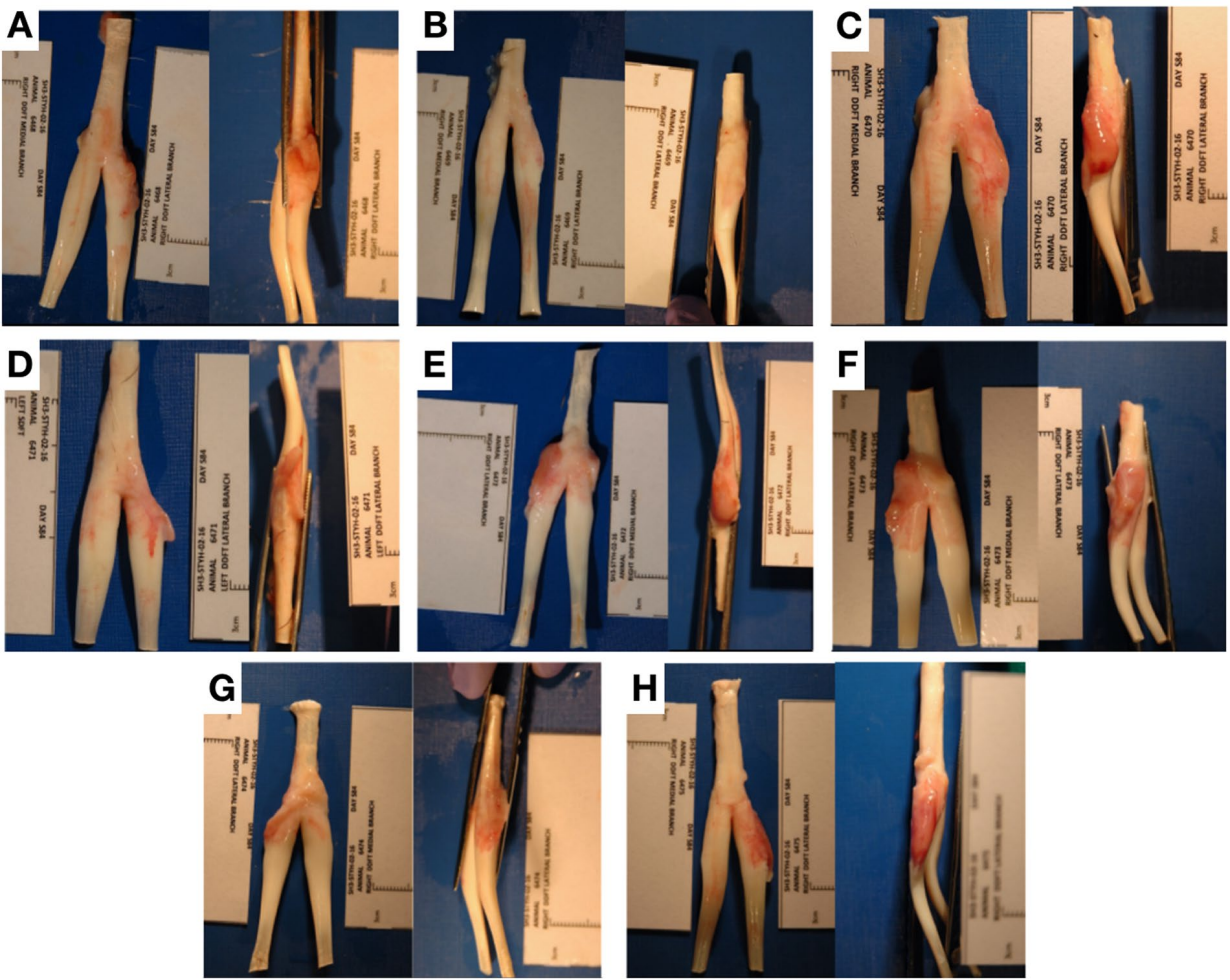
Digital photograph images showing harvested tendons augmented with patch scaffolds. Proximal is to the top of the image.

Scanning electron microscopy of the electrospun layer demonstrated aligned fibers in a crimp morphology. The median fiber diameter of the 980 nm for the electrospun layer (Interquartile range, IQR 802–1323 nm). The woven PDO layer demonstrated a twill weave pattern as expected. Median fiber diameter was 118μm (IQR 114–134μm) (Fig. 3).

### Macroscopic observations of electrospun scaffold implanted tendons

There was no difference in the circumference of the forelimb around the metacarpophalangeal joint MCPJ (above and below the ergot) measured pre-surgery and 3 months later, in all sheep. Macroscopic inspection of the lateral branch of the deep digital flexor tendon after removal of the tendon demonstrated healing of the defect in all sheep 3 months after patch implantation. There was no residual polymer from the electrospun scaffolds seen on the surface of the tendon in any specimen.

**Figure 4:**
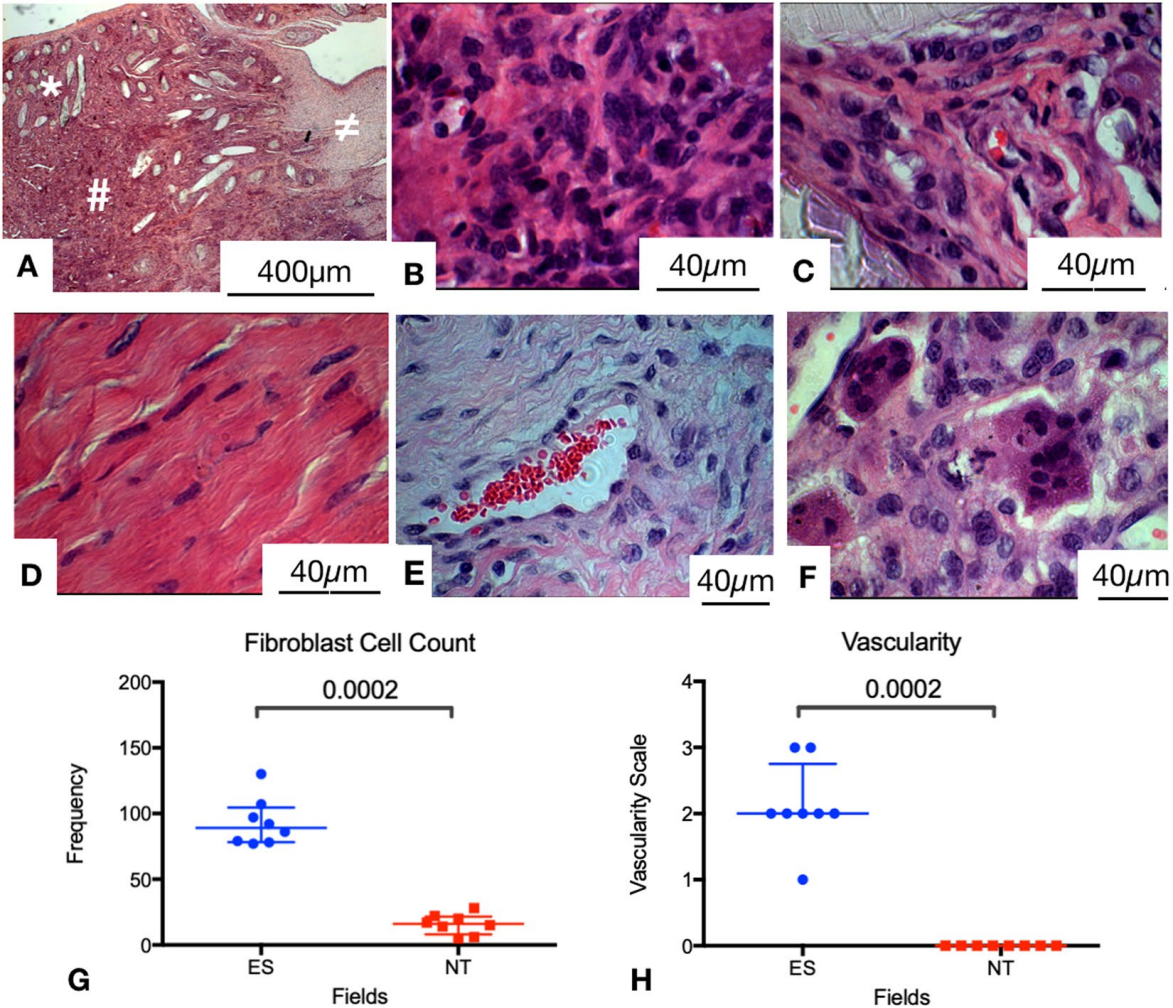
(**A**) Low magnification (2.5X) bright field microscopy image of H&E staining showing woven PDO zone (*), electrospun PDO zone (#), and normal tendon zone (≠) of patch augmented repair B-D: High magnification bright field microscopy histology (H&E staining) images of patch augmented tendon repair. For the patch images, 3 fields shown are electrospun fiber section (**B**), section adjacent to woven PDO monofilament (**C**), and normal tendon section (**D**). (**E**) Representative high magnification (100X) bright field microscopy histology (H&E staining) image demonstrating blood vessels within patch augmented tendon. (**F**) Representative high magnification (100X) bright field microscopy histology (H&E staining) image demonstrating foreign body giant cells in patch augmented tendon. (**G**,**H**) Quantitative histology results for patch samples. Fibroblast cell count number by zones imaged (**G**). Vascularity grade by zone (**H**). ES = electrospun fiber zone; NT = normal tendon zone. P values (Mann Whitney U test) given above compared fields.

A smooth pink-red layer, superficial to the tendon, overlying the healed defect, was present. This layer was indistinct from the underlying tendon and appeared to be fully integrated with host tissue. It appeared softer, and more vascular than the tendon. This layer was rarely adherent to the surrounding sheath. When mild adhesions were observed, the DDFT was easily separated from the surrounding sheath without the need for sharp dissection. There was minimal (<0.5 ml) synovial fluid which had a normal appearance (clear, straw-colored, viscous fluid), and a mild local inflammatory reaction in the surrounding synovium (Fig. 4).

### Hematology and serum inflammatory markers of scaffold implanted sheep

Hematology testing of all sheep for full blood count (including white cell count, neutrophil count, lymphocyte count, and red blood cell count) demonstrated no significant change. Additionally, serum markers for inflammation (serum amyloid A and fibrinogen) were not elevated at 3 months post-surgery compared to pre-surgery levels in all sheep.

### Histology of electrospun scaffold implanted tendons

Hematoxylin and eosin (H&E) staining demonstrated several key features. The electrospun scaffolds were present in an area of increased cellularity and vascularization. Each section consistently demonstrated three distinct areas (Fig. 4A). The most superficial layer contained remnants of woven PDO monofilament. Residual polymer from the PCL layers were not consistently identified due to sectioning the tendon explants longitudinally. Continuous and deep to this layer was the electrospun PDO layer. This area was occupied by a large number of cells and demonstrated neovascularization. Remnant PDO material was rarely seen in this zone at high (100X) magnification. The deepest layer of the section represented the native sheep tendon underlying the patch. These areas had similar appearance to a normal tendon, but with fewer fibrils with the characteristic crimp morphology of normal tendon (Fig. 4D).

In both electrospun scaffold zones, scant areas of foreign body giant cells were seen (Fig. 4F). A network of blood vessels supported the tissue integrated into the electrospun scaffolds (Fig. 4E). The neovascularization observed was seen within the patch scaffold.

The electrospun fiber zones were significantly different in terms of fibroblast cell count (median 89, 95% CI 78–108 vs 16, CI 9–22, p = 0.0002), and vascularity (median vascularity grade 2, CI 1.6–2.7 vs 0, p = 0.0002) compared to the normal tendon zones. There are no differences in FBGC count in the electrospun fiber and normal tendon zones as these were rarely seen in either zone (median 0 FBGCs seen in both NF and NT zones). Neutrophils, which can be identified by H&E stainining based upon their distinct morphology were not identified in scaffold implanted ovine tendons.

### Immunohistochemistry

**Figure 5:**
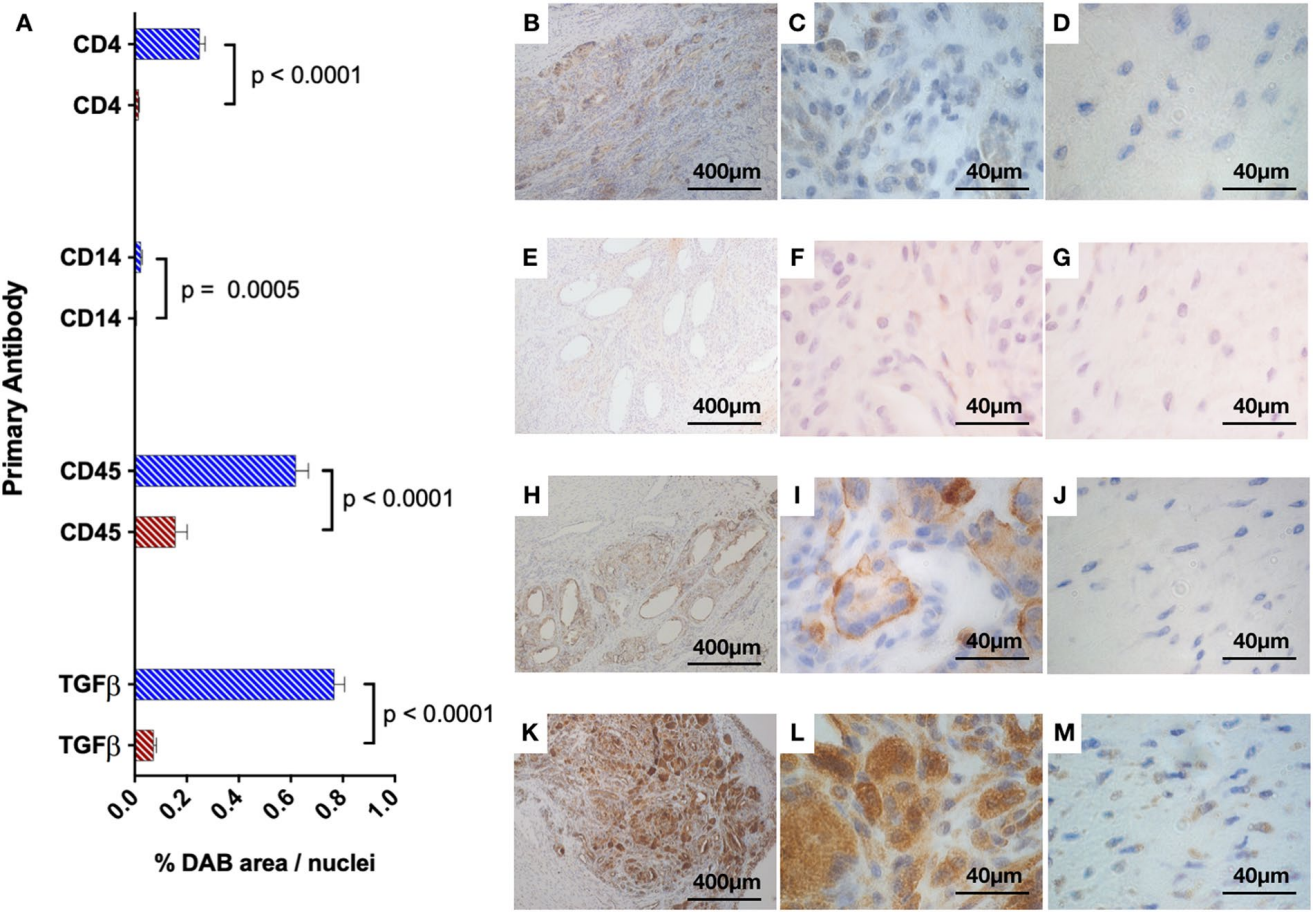
(**A**) Quantitative immunohistochemistry (IHC) results for patch samples. Red bars = Normal Tendon (NT) zone. Blue bars = Electrospun fiber (ES) zone. Percentage area of DAB stain per nuclei for primary antibodies in zones imaged. ES = electrospun fiber zone; NT = normal tendon zone. P values (Mann Whitney U test) provided to the right of compared fields. Representative images showing immunohistochemistry (IHC) for anti-CD4, anti-CD14, anti-CD45, and anti-TGFβ. (**B**,**E**,**H**,**K**) low magnification (10X) bright field images showing both ES and NT zones. (**C**,**F**,**I**,**L**) images taken at high magnification (100X) in the electrospun fiber (ES) zones. (**D**,**G**,**J**,**M**) images taken at high magnification (100X) in the normal tendon (NT) zones.

There were significantly increased numbers of CD4^+^, CD45^+^, and CD14^+^ cells in the electrospun fiber zones compared to the normal tendon zones (p < 0.0001, p < 0.0001, and p = 0.0005 respectively). However, fewer CD14^+^ cells were identified than CD4^+^ cells. Immune cells were uniformly distributed throughout the electrospun patch. TGFβ immunopositive staining was markedly increased in the electrospun fiber zones compared to the normal tendon zones (p < 0.0001) (Fig. 5).

Given the high number of fibroblasts (characterized by their large nuclei and small cytoplasm), Collagen I and III staining was assessed^19^. Within the normal tendon zones, Collagen I subtype α2 was observed however, in the electrospun fiber zones, more intense immunopositive staining was seen. For Collagen III subtype α1, no staining was present in the ECM of the normal tendon zones, however, the electrospun fiber zones showed immunopositive staining. The presence of Collagen III in the tissue surrounding the patch is consistent with fibrous tissue healing.

### Adverse events

There were no adverse events noted. All sheep resumed normal activity after scaffold implantation. There was no evidence of lameness in any sheep after surgery.

## Discussion

This *in vitro* study used a sheep tendon injury model characterized by a natural history of non-healing. In particular recent work has shown that such lesions treated with autologous mesenchymal stem cells did not show any evidence of tissue healing [20]. Here we show that the implantation of a woven electrospun PDO/PCL scaffold promoted an endogenous tendon fibroblast response and evidence of tendon healing. The electrospun component of the scaffolds integrated with host tendon tissue in all sheep with minimal external signs of inflammation and without significant adhesion formation to the surrounding tissues. We observed significant ingrowth and proliferation of endogenous cells into the electrospun fibre, but not the woven, component of the patch indicating that the electrospun component is providing cues that promote alteration in cell behavior. We also observed significant new blood vessel development within the electrospun part of the scaffold. A mild local inflammatory response was seen 3 months after surgery which was consistent with the expected host response to a biodegradable polymer. Some residual woven monofilament PDO was seen in 5 of 8 sheep at 3 months and, in contrast to the electrospun component, demonstrated no cellular infiltration. The predominant cell type that infiltrated the electrospun layers were tendon fibroblasts exhibiting a high nuclear:cytoplasmic ratio^19^. No systemic inflammatory response was detected by hematology and serology. There were no signs of infection or tumor formation.

Compared to biological scaffolds currently being proposed for rotator cuff tendons, this PDO/PCL scaffold has the advantage to be fully synthetic. This reduces the risk of immunogenicity of the materials and enables consistent manufacturing (no batch to batch variation). Compared to other synthetic materials, it has the advantage of being fully degradable and to have both a bioactive component (electrospun layers) and mechanical component (woven reinforcement layer). These advantages have been discussed in a previous publication^13^.

Several studies have implanted electrospun polymer scaffolds in various small and large animal models showing significant fibroblast infiltration without any evidence of an adverse foreign body reaction^20–23^. Implanting random and aligned electrospun PLLA scaffolds into two murine models, Yin *et al*. demonstrated complete infiltration of spindle shaped cells along the nanofibers by 6 weeks^22^.

Our study demonstrates neovascularization of collagen-containing tissue produced by fibroblasts in the electrospun polymer layers. This mild and evenly distributed proliferation of vessels within the electrospun component may provide a positive and useful component of the new tissue formation, supportive to tendon healing. The observation of a rich network of new blood vessels has been a key finding in other *in vivo* applications of electrospun scaffolds. Implanted electrospun polycaprolactone-gelatin composites in a rat bone defect model demonstrated a thin fibrous capsule with neovascularization of the scaffolds^24^. Whilst no other study has shown neovascularization in tendon augmentation with an electrospun PDO/PCL patch. Several studies have successfully used electrospun PDO and PDO/PCL scaffolds as vascular grafts, which demonstrated patent, neovascularized, and epithelialized tissue^25–27^. Staining for CD45^+^ and CD4^+^ cells was also increased compared to the normal tendon zones. CD14^+^ cells, were significantly increased in the electrospun fiber zones compared to the normal tendon zones, however they were detected in much smaller levels than CD45^+^ and CD4^+^ cells. Immune cells are required to degrade the polymer material as well as stimulate tendon stromal cells to produce ECM components. Other authors have also reported increased CD45 expression using a twisted yarn of electrospun PCL fibers^28^.

We previously identified macrophage phenotype is complex and plastic in human tendon disease, demonstrating macrophages exhibit interferon, NF- κB, STAT-6 and glucocorticoid receptor activation states^29,30^. This necessitates a cassette or panel or markers to comprehensively and robustly characterise macrophage activation states. This is readily accomplished in human tissues, where most antibodies cross react with human proteins. We tested a panel of macrophage markers routinely used to characterise human macrophage activation states in ovine tissues, and found that these antibodies did not cross react with ovine proteins. We were unable to successfully source commercially available antibodies for macrophage markers that cross reacted with ovine tissues, hence we cannot comment upon the phenotypes of macrophages populating in implanted ovine tendons in this study.

The strengths of this study is that we used a large animal non-healing model of tendon injury to assess efficacy. We are not aware of any other small or large animal models of tendon injury where the injury consistently fails to heal without intervention. Limitations include the single time point used for tendon harvest. Normal tendon was used as the control comparator along with control data from the Khan study [21]. The lack of reliable and specific cell markers for ovine tendon fibroblasts necessitated a reliance on identification using cell histomorphometry. Due to the nature of the tendon injury model as a longitudinal split, this non-healing model could not test the ability to repair the tendon under relevant tensile mechanical loading, which was an important limitation. However, we believe that the biological process behind the healing was a more important issue to address in the context of rotator cuff tendon repair (it is thought to be a major reason for failure of current repairs) and so far existing *in vivo* models have failed to provide a non-healing model of tendon (i.e. they tend to repair even without surgical intervention). Furthermore, because of our focus on the biological response to the electrospun scaffold and because of the limited number of samples available, we have not performed mechanical testing of the healed longitudinal tendon tissue.

## Conclusions

The electrospun fiber component of a laminated patch induced an endogenous, predominantly tendon fibroblast, cellular response and enabled tendon healing in an ovine model without evidence of abnormal local or systemic foreign body response.
